# Antibody Titer Correlates with Omicron Infection in Vaccinated Healthcare Workers

**DOI:** 10.3390/v14122605

**Published:** 2022-11-23

**Authors:** Maximiliano Mollura, Riccardo Sarti, Riccardo Levi, Chiara Pozzi, Elena Azzolini, Letterio S. Politi, Alberto Mantovani, Riccardo Barbieri, Maria Rescigno

**Affiliations:** 1 Department of Electronic, Information and Bioengineering, Politecnico di Milano, Piazza Leonardo da Vinci 32, 20133 Milano, Italy; 2 Department of Biomedical Sciences, Humanitas University, Via Rita Levi Montalcini, Emanuele, 20072 Milan, Italy; 3 IRCCS Humanitas Research Hospital, Via Manzoni 56, Rozzano, 20089 Milan, Italy; 4 The William Harvey Research Institute, Queen Mary University of London, London E1 4NS, UK

**Keywords:** COVID-19, SARS-CoV-2 Omicron variant, vaccine, IgG

## Abstract

The advent of vaccines against SARS-CoV-2 has drastically reduced the level of hospitalization with severe COVID-19 disease in infected individuals. However, the diffusion of variants of concern still challenge the protection conferred by vaccines raised against the wild-type form of the virus. Here, we have characterized the antibody response to the BNT162b2 (Comirnaty) mRNA vaccine in patients infected with the Omicron variant. We analyzed a population of 4354 vaccinated healthcare workers (HCW) from 7 different hospitals in Italy and monitored infection with SARS-CoV-2 Omicron. We correlated infection with the antibody response after vaccination. We found that a lower level of IgG, younger age, and the presence of allergies correlate with increased infection during the Omicron wave, and that infections correlate with wild-type spike protein antibody titers below 350 BAU/mL. These results support the necessity of a fourth booster dose, particularly for individuals with lower levels of antibodies.

## 1. Introduction

Vaccination against SARS-CoV-2 has drastically impacted viral diffusion during the course of the pandemic, saving millions of lives [1,2,3]. Vaccine formulations—whether they are mRNA, DNA or protein based—have been directed to the spike protein of the wild-type Wuhan variant of the virus [4,5]. However, several variants of concern (VOC) have been emerging in the last two years [6]. In Italy, there was a first wave between February and September 2020 with the wild-type variant, a second wave between October 2020 and July 2021 with the B.1.1.7 (Alpha) variant [7], and a third wave between August 2021 and March 2022 characterized by B.1.617.2 (Delta) variant [8] at first, but was then quickly surmounted by B.1.1.529 (Omicron) variant [9]. Omicron BA.1, and even more so BA.4 and BA.5, have many different mutations, particularly in the Spike protein [10] which has been used to generate most of the recombinant vaccines. Nevertheless, the vaccine has been shown to also generate neutralizing antibodies to the Omicron VOC, however with different efficiency among the vaccinated population [11]. While the protection against severe disease caused by Delta variant is around 84.9% to 90.3%, the effect against Omicron drops to a range between 56.5% to 82.4% [12]. Therefore, an important question is whether there is a level of antibodies correlating with protection against disease from the very contagious Omicron VOC. We analyzed the entire population of 4354 healthcare workers (HCW) from 7 different humanitas hospitals in Lombardy, Italy. HCW were monitored for the development and duration of the immune response after vaccination and for infection with SARS-CoV-2. We found that a lower level of IgG, younger age, and the presence of allergies correlated with increased infection during the Omicron wave. Interestingly, infections correlated with antibody titers below 350 BAU/mL.

## 2. Materials and Methods

This is a longitudinal study on 4354 healthcare workers (HCW) from 7 different healthcare facilities in Lombardy, Italy. Subjects were vaccinated with the first two doses of BNT162b2 (Comirnaty) mRNA vaccine in the period of January and March 2021. Each subject underwent blood sample for quantitative anti-SARS-CoV-2 IgG serum level between 9 September and 7 October 2021 (6–8 months after the second dose), performed with LIAISON SARS-CoV-2 TrimericS IgG (DiaSorin), a quantitative CE-marked assay for the detection of IgG antibodies recognizing the native trimeric Spike glycoprotein of SARS-CoV-2 [13]. The levels of IgG antibodies are expressed in Binding Antibody Units per milliliter (BAU/mL). Samples ≥ 33.8 BAU/mL were considered positive according to the technical documentation of the manufacturer. Each participant received a third dose of the vaccine between September 2021 and February 2022, and was asked to fill in a survey including demographics, comorbidities, SARS-CoV-2 related symptoms and their duration, and vaccination status between February and March 2022.

After considering all the variables of interest (Sex, Age, BMI and COVID-19 history), the compilation of the questionnaire and the administration of a third dose of vaccine, we analyzed 2329 subjects of which 288 (12.4%) with a SARS-CoV-2 infection between 1 January 2022 and 1 March 2022 (Omicron wave).

The descriptive information of the study cohort is reported in Table 1. We used the χ^2^ test to evaluate the association between categorical variables, *t*-test (two-sided) or Mann–Whitney test (two-sided) were applied in normally or non-normally distributed continuous variables, respectively (Shapiro–Wilk test, significance *p* < 0.05). Significance threshold was set to 0.05.

In order to test the association between IgG levels and risk of getting infected during the Omicron wave, we evaluated a multivariate logistic regression model including measured IgG and sex, age, history of SARS-CoV-2 infection, allergies, body mass index (BMI), and the days between IgG sampling and third dose of vaccination as possible confounding variables. IgG levels, age, and BMI variables were standardized using Z-transformation, resulting in null mean and unitary standard deviation. Therefore, odds ratios for IgG levels, age, and BMI are related to a unitary standard deviation increase, respectively equal to 639 BAU/mL, 12 years and 4 kg/m^2^. Reference baseline model consists of female subjects, without allergies and without any history of previous SARS-CoV 2 infections, at average age (45 y.o.), BMI (23.8 kg/m^2^), and IgG levels (626 BAU/mL). Finally, we repeatedly tested the association of several thresholds of IgG moving from 100 BAU/mL to 2000 BAU/mL with step equal to 50 BAU/mL to have an estimate of the optimal threshold of IgG associated with the infection (significance was set to *p* < 0.01 to account for multiple testing).

## 3. Results and Discussions

We analyzed the occurrence of SARS-CoV-2 Omicron infection in relation to IgG levels when accounting for sex, age, history of SARS-CoV-2 infection, allergies, BMI, and for the days between IgG sampling and administration of the third dose (Table 1). On average, subjects tested positive to SARS-CoV-2 after 69 (±21) days after the administration of the third dose. Univariate testing of infection showed significant association with age (*p* < 0.001), IgG level (*p* = 0.003), allergy (*p* = 0.004), and history of COVID-19 (*p* = 0.032).

**Figure 1:**
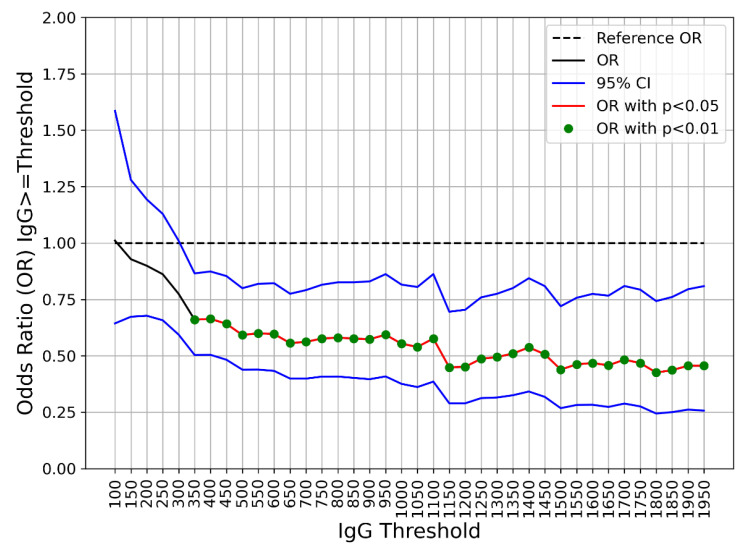
Odds ratios (OR) (black line) and 95% confidence interval (blue lines) obtained while varying the IgG threshold (BAU/mL) and testing its association with an Omicron infection. The red line and green dots represent the odds significantly different from 1 (dashed line), respectively, with *p* < 0.05 and *p* < 0.01.

From the logistic regression analysis (Table 2), the presence of allergies (OR 1.40, 95% CI 1.08–1.80, *p* = 0.010), age (OR 0.75, 95% CI 0.66–0.86, *p* < 0.001), and IgG level (OR 0.74, 95% CI 0.62–0.87, *p* < 0.001) were strongly associated with the Omicron infection. The repeated logistic regression analysis including different thresholds of IgG levels shows significant associations (*p* < 0.01) starting from IgG ≥ 350 BAU/mL with odds ratios consistently and significantly smaller than 1, as shown in Figure 1.

These results support previous data showing that the vaccine against the wild-type version of the SARS-CoV-2 virus induces antibodies capable of neutralizing even the Omicron VOC [11,14,15,16,17] which carries more than 30 new mutations. We also show that infections during the Omicron wave occur in individuals with level of circulating anti spike IgG antibodies below 350 BAU/mL. Not surprisingly, this number is higher than the one we found during the previous SARS-CoV-2 VOC infections (around 100 BAU/mL) [18]. Indeed, in another study, it has been shown that the level of neutralizing antibodies, after vaccination with the wild-type SARS-CoV-2-directed mRNA vaccines, to Non-Omicron SARS-CoV-2 VOC is very similar to that towards the wild-type variant with a fold reduction of only 2.8 for Alpha, 6.9 for Beta, 5.4 for Gamma, 3.5 for Delta, 4.3 for Zeta, while that to Omicron was much lower (85.7 fold reduced) with 30% of individuals with no neutralizing antibodies to Omicron at all [11]. Of note, in the latter study the tests were performed on sera collected 30 days from the second vaccination dose, thoughit has been shown that the highest protection against symptomatic omicron infection is obtained after the third dose [17,19]. Indeed, a recent paper has reported that individuals boosted with the third dose of mRNA-based COVID-19 vaccine display potent neutralization of Omicron, only 4–6 fold lower than the wild-type [17]. To this extent, the chances of encountering neutralizing antibodies to Omicron after two doses of the vaccine is low, and in individuals with a higher serum level of antibodies, the chance of having developed antibodies to the Omicron VOC increases. Our results suggest that individuals with levels of anti-spike IgG antibodies below 350 BAU/mL, as measured with our test, have higher risk of infection despite having received the third dose, suggesting that below 350 BAU/mL of anti-spike IgG antibodies it is unlikely to find anti-Omicron neutralizing antibodies capable to protect from infection. Consequently, an increase of antibody levels via an additional booster dose would be important to avoid infection regardless of its tailoring towards a specific variant. Indeed, if it is confirmed that the new variant-modified COVID-19 vaccine boosters do not seem to favor the induction of an immune response specific against Omicron VOC, any fourth dose of the COVID-19 vaccine would still be desirable [20].

It should also be noted that 350 BAU/mL should not be considered as a threshold for vaccinating individuals, as other VOC may arise in the meantime. Indeed, in a previous study on immunocompromised individuals, we identified a protective threshold of 100 BAU/mL, which was related to a different VOC [18]. The continuous evolution of new VOC makes it particularly difficult to identify a threshold of protection as it varies according to the circulating virus, and to quantify the individual chances of having generated a more extensive repertoire which covers also new VOC.

The main limitation of this study is the assumption that during the period of analysis all the infections were due to Omicron. However, this is a controlled guess, as the Italian observatory of COVID-19 infections has shown that Omicron was the primary VOC (>88%) in the period of analysis.

Finally, we tested antibodies to the wild-type form of the SARS-CoV-2 spike protein as these are induced with the vaccine. A test directed to the spike protein of the Omicron may most likely highlight different antibody levels correlating with infection.

## Figures and Tables

**Table 1:** Demographic information of the study population.

	Overall	Omicron Infection	Non-Omicron Infection	*p*-Value
Total	2324	288	2036	
Males (n, %)	638 (27.5%)	65 (22.6%)	573 (28.1%)	0.056
Females (n, %)	1686 (72.5%)	223 (77.4%)	1463 (71.9%)	
Age (mean, SD)	45.3 (12.7)	42.6 (11.0)	45.7 (12.7)	**<0.001**
Body Mass Index (mean, SD)	23.8 (4.0)	23.4 (3.7)	23.9 (4.1)	0.076
IgG level (mean, SD)	626.5 (639.0)	482.4 (502.3)	646.9 (653.6)	**0.003**
Allergy (n, %)	783 (33.7%)	119 (41.3%)	664 (32.6%)	**0.004**
Previous SARS-CoV-2 Infection (n, %)	398 (17.1%)	36 (12.5%)	362 (17.8%)	**0.032**
Days between IgG sampling and 3rd dose (mean, SD)	49.5 (18.4)	47.5 (16.5)	49.8 (18.7)	0.129

**Table 2:** Results of multivariate logistic regression for Omicron Infection.

Features	Odds Ratio (95% CI)	*p*-Value
Intercept	0.16 (0.11–0.25)	**<0.001**
Sex	0.76 (0.56–1.03)	0.079
Age	0.75 (0.66–0.86)	**<0.001**
Body Mass Index	0.97 (0.85–1.11)	0.633
Allergy	1.40 (1.08–1.80)	**0.010**
IgG level	0.74 (0.62–0.87)	**<0.001**
Previous SARS-CoV-2 Infection	0.85 (0.57–1.26)	0.416
Days between IgG sampling and 3rd dose	0.99 (0.99–1.00)	0.194

## Data Availability

According to the policy of the hosting Institution, data will be made available through Zenodo.

## References

[1] Watson O.J., Barnsley G., Toor J., Hogan A.B., Winskill P., Ghani A.C. (2022). Global impact of the first year of COVID-19 vaccination: A mathematical modelling study. Lancet Infect. Dis..

[2] Barouch D.H. (2022). COVID-19 Vaccines—Immunity, Variants, Boosters. N. Engl. J. Med..

[3] Rojas-Botero M.L., Fernández-Niño J.A., Arregocés-Castillo L., Ruiz-Gómez F. (2022). Estimated number of deaths directly avoided because of COVID-19 vaccination among older adults in Colombia in 2021: An ecological, longitudinal observational study. F1000Research.

[4] Patel R.S., Agrawal B. (2022). Heterologous immunity induced by 1st generation COVID-19 vaccines and its role in developing a pan-coronavirus vaccine. Front. Immunol..

[5] Jeyanathan M., Afkhami S., Smaill F., Miller M.S., Lichty B.D., Xing Z. (2020). Immunological considerations for COVID-19 vaccine strategies. Nat. Rev. Immunol..

[6] Choi J.Y., Smith D.M. (2021). SARS-CoV-2 Variants of Concern. Yonsei Med. J..

[7] Shen X., Tang H., McDanal C., Wagh K., Fischer W., Theiler J., Yoon H., Li D., Haynes B.F., Sanders K.O. (2021). SARS-CoV-2 variant B.1.1.7 is susceptible to neutralizing antibodies elicited by ancestral spike vaccines. Cell Host Microbe.

[8] Kannan S.R., Spratt A.N., Cohen A.R., Naqvi S.H., Chand H.S., Quinn T.P., Lorson C.L., Byrareddy S.N., Singh K. (2021). Evolutionary analysis of the Delta and Delta Plus variants of the SARS-CoV-2 viruses. J. Autoimmun..

[9] Hui K.P.Y., Ho J.C.W., Cheung M.-C., Ng K.-C., Ching R.H.H., Lai K.-L., Kam T.T., Gu H., Sit K.-Y., Hsin M.K.Y. (2022). SARS-CoV-2 Omicron variant replication in human bronchus and lung ex vivo. Nature.

[10] Arora S. (2022). Omicron: A variant of concern not a cause of panic. J. Adv. Pharm. Technol. Res..

[11] Bekliz M., Adea K., Vetter P., Eberhardt C.S., Hosszu-Fellous K., Vu D.-L., Puhach O., Essaidi-Laziosi M., Waldvogel-Abramowski S., Stephan C. (2022). Neutralization capacity of antibodies elicited through homologous or heterologous infection or vaccination against SARS-CoV-2 VOCs. Nat. Commun..

[12] Shao W., Chen X., Zheng C., Liu H., Wang G., Zhang B., Li Z., Zhang W. (2022). Effectiveness of COVID-19 vaccines against SARS-CoV-2 variants of concern in real-world: A literature review and meta-analysis. Emerg. Microbes Infect..

[13] Bonelli F., Blocki F.A., Bunnell T., Chu E., De La O.A., Grenache D.G., Marzucchi G., Montomoli E., Okoye L., Pallavicini L. (2021). Evaluation of the automated LIAISON^®^ SARS-CoV-2 TrimericS IgG assay for the detection of circulating antibodies. Clin. Chem. Lab. Med..

[14] Arbel R., Sergienko R., Friger M., Peretz A., Beckenstein T., Yaron S., Netzer D., Hammerman A. (2022). Effectiveness of a second BNT162b2 booster vaccine against hospitalization and death from COVID-19 in adults aged over 60 years. Nat. Med..

[15] Cohen M.J., Oster Y., Moses A.E., Spitzer A., Benenson S., Abu-Ahmad A., Angel Y., Ben-Ami R., Ben-David D., Israeli-Hospitals 4th Vaccine Working Group (2022). Association of Receiving a Fourth Dose of the BNT162b Vaccine With SARS-CoV-2 Infection Among Health Care Workers in Israel. JAMA Netw. Open.

[16] Muhsen K., Maimon N., Mizrahi A.Y., Boltyansky B., Bodenheimer O., Diamant Z.H., Gaon L., Cohen D., Dagan R. (2022). Association of Receipt of the Fourth BNT162b2 Dose With Omicron Infection and COVID-19 Hospitalizations Among Residents of Long-term Care Facilities. JAMA Intern. Med..

[17] Garcia-Beltran W.F., St Denis K.J., Hoelzemer A., Lam E.C., Nitido A.D., Sheehan M.L., Berrios C., Ofoman O., Chang C.C., Hauser B.M. (2022). mRNA-based COVID-19 vaccine boosters induce neutralizing immunity against SARS-CoV-2 Omicron variant. Cell.

[18] Azzolini E., Pozzi C., Germagnoli L., Oresta B., Carriglio N., Calleri M., Selmi C., De Santis M., Finazzi S., Carlo-Stella C. (2022). mRNA COVID-19 vaccine booster fosters B- and T-cell responses in immunocompromised patients. Life Sci. Alliance.

[19] Accorsi E.K., Britton A., Fleming-Dutra K.E., Smith Z.R., Shang N., Derado G., Miller J., Schrag S.J., Verani J.R. (2022). Association Between 3 Doses of mRNA COVID-19 Vaccine and Symptomatic Infection Caused by the SARS-CoV-2 Omicron and Delta Variants. JAMA.

[20] Khoury D.S., Docken S.S., Subbarao K., Kent S.J., Davenport M.P., Cromer D. (2022). Predicting the efficacy of variant-modified COVID-19 vaccine boosters. medRxiv.

